# Effects of early life adversity and sex on dominance in European starlings

**DOI:** 10.1016/j.anbehav.2017.03.026

**Published:** 2017-06

**Authors:** Tom Bedford, Caitlin Jade Oliver, Clare Andrews, Melissa Bateson, Daniel Nettle

**Affiliations:** Centre for Behaviour and Evolution & Institute of Neuroscience, Newcastle University, Newcastle, UK

**Keywords:** birds, dominance, early life adversity, individual quality, resource competition, starlings

## Abstract

Dominance in socially foraging animals may be related to sex and to variation in individual quality. Individual quality may in turn reflect conditions during early development. We studied dominance in a cohort of adult European starlings, *Sturnus vulgaris*, that had been subject to experimental manipulations of food supply and begging effort when they were nestlings. We measured dominance in two different contexts, contests over a food resource and relative position on a sloping perch, over the course of 3 weeks. Dominance in food contests was extremely stable over the 3 weeks and relative perch position somewhat stable. Males were dominant over females in contests over food and perched in higher positions. These sex differences were not explained by males' greater size or body weight. Food dominance and perch position were uncorrelated. Neither early life food supply nor early life begging effort affected food dominance; nor did an alternative measure of developmental stress, developmental telomere attrition. Birds that had been made to beg more as nestlings perched in higher positions than those that had begged less. Our results did not support the hypothesis that early life adversity leads to lower adult dominance rank in the context of feeding, and we suggest that relative perch position may have measured individual preference rather than competitive ability.

## Highlights

• Dominance in social animals may be affected by early life conditions.
• We studied a cohort of starlings of known developmental histories.
• We measured perching position and dominance in food contests.
• Males perched above females and were more dominant.
• Developmental history affected perch position but not dominance.

Socially foraging animals of many species form dominance hierarchies in which each individual can be characterized by a social rank (Chase, Tovey, Spangler-Martin, & Manfredonia, 2002). Dominant or higher-ranking individuals are able to displace subordinate or lower-ranking individuals from sought-after resources, and dominance is typically positively related to reproductive success (Ellis, 1995). Attained positions in dominance hierarchies are often, although imperfectly, related to variation in individual attributes such as size, age or individual quality (Chase et al., 2002). For example, in birds, dominance rank often correlates with plumage ornamentation, which, in both sexes, can function as an indicator of quality (Rat et al., 2015, Santos et al., 2011, Senar et al., 1993, Swaddle and Witter, 1995).

Individual quality may partly reflect genetic variation (Ryder, Tori, Blake, Loiselle, & Parker, 2010), but it is also likely to be influenced by developmental history. The evidence for developmental history affecting dominance rank is indirect. In passerine birds, dominance has been shown to correlate with traits such as song repertoire size (Spencer, Buchanan, Goldsmith, & Catchpole, 2004); song repertoire size in turn has been shown to reflect developmental history, with more adverse histories leading to smaller song repertoires (Buchanan et al., 2003, Nowicki et al., 2002). Thus, the hypothesis that adverse developmental experience would lead to lower dominance rank in adulthood is a reasonable one. It has not, however, been directly tested experimentally.

In recent studies in the European starling, *Sturnus vulgaris*, we have found that individuals that experience more early life adversity (due to competition during the nestling phase) show a number of behavioural differences in adulthood from those that experience less adversity. For example, they are less selective in what they eat (Bloxham, Bateson, Bedford, Brilot, & Nettle, 2014), and they tend to be hyperphagic and heavier for their skeletal size (Andrews et al., 2015). Accumulating fat reserves is a strategy used by subordinate individuals in the starling (Witter & Swaddle, 1995), and other passerines (Ekman & Lilliendahl, 1993), as insurance against their risk of exclusion from food resources. The reduced dietary selectivity might also reflect a foraging strategy appropriate for subordinate individuals, which may be restricted to lower-preference food resources and at risk of displacement from feeding sites by dominants. Thus, one simple explanation for the behavioural differences we have found between adults that experienced early life adversity and those that did not is that the early adversity birds are socially subordinate as adults, and therefore display the behavioural traits typical of subordinates. This explanation garners plausibility from the fact that forms of early life deprivation and insult have been shown to predict subordinate status in adulthood in primates and rodents (Bastian et al., 2003, Benner et al., 2015). To establish whether the explanation is correct, it is first necessary to determine whether early life adversity does indeed affect adult dominance in the starling.

In the present study, we investigated the relationships between early developmental history and adult dominance in a cohort of starlings previously hand-reared and subjected to an early adversity manipulation (Nettle et al., 2017). The 32 birds consisted of eight sets of four siblings. Within each sibling set, one bird was assigned to each of four experimental groups from day 5 to day 15 posthatching. The groups were composed of the factorial combinations of a high or a low amount of available food (henceforth, Plenty versus Lean Amount treatments), and a high or a low level of begging required per day (henceforth, Hard versus Easy Effort treatments). The four experimental groups were thus: Plenty-Easy, Plenty-Hard, Lean-Easy and Lean-Hard. After day 15, experimental groups were mixed together and kept in uniform conditions until adulthood. We then assembled them into their sibling quartets to measure dominance relative to their siblings from the other experimental groups at around 1 year old.

Our main question of interest was whether the experimental treatments (Amount and Effort) predicted dominance. We also aimed to test whether the attrition of erythrocyte telomeres over the developmental period (days 5–56) predicted dominance. Telomeres are the repetitive DNA caps on the ends of chromosomes that shorten with age, and the extent of developmental telomere attrition has been proposed as an integrative measure of the impact of developmental stressors (Boonekamp et al., 2014, Herborn et al., 2014). The experimental treatments both affected developmental telomere attrition, and did so additively (Nettle et al., 2017). However, developmental telomere attrition potentially provides a better measure of developmental stress than experimental treatment. First, developmental telomere attrition may capture variation within experimental groups in how severe the impact of the treatment was. Second, developmental telomere attrition also integrates sources of developmental stress other than those stemming from our experimental design. In several previous studies we have found that developmental telomere attrition is a better predictor of adult phenotype than experimental group (Bateson et al., 2015, Nettle et al., 2015). Thus, a secondary objective was to test the possibility that developmental telomere attrition would be an additional, or perhaps better, predictor of adult dominance than the experimental treatments.

Previous studies of dominance in the European starling have performed one or more bouts of observation on a set of birds, and used the outcomes of all the competitive interactions over resources to derive a single dominance score for each individual within the group (Feare and Inglis, 1979, Spencer et al., 2004, Swaddle and Witter, 1995, Witter and Swaddle, 1995). Thus, these studies assume that dominance within a given group is stable over time, so that either a single observation bout suffices, or that data from different times can be amalgamated. However, no study has tested the assumption of stability over time. Moreover, while the contested resource is usually food, one study also included competitive interactions over perching sites in the calculation of dominance (Swaddle & Witter, 1995). Combining food and perch contests into a single dominance score assumes that the dominance ranking is the same for these two situations. This is an assumption that is untested in the starling, although there is evidence from the chaffinch, *Fringilla coelebs*, that food and perch contests produce the same hierarchy (Marler, 1955). Given that early life adversity may change the motivational salience of food (Andrews et al., 2015, Bloxham et al., 2014), it was particularly important for our current purposes to be able to separate out dominance in food-related interactions from those in other contexts, as the experimental treatments may have affected each in different ways. Thus, in our study we aimed to measure dominance in food contests separately from the ability to defend a preferred perch position; and to measure dominance in both contexts over 3 successive weeks, to establish whether each of these rankings was in fact stable over time.

The predictions of our study were as follows. First, we predicted that both dominance in competition over a valued food resource (since this is the typical way dominance is measured, we henceforth refer to it simply as ‘dominance’) and ability to defend a desirable perch position (henceforth ‘perch position’) would show stability over time, with scores in successive weeks correlated with one another. Second, we predicted that dominance and perch position would be correlated with one another, on the assumption that both reflect the same underlying ranking of competitive ability. Third, we predicted that greater early life adversity (either Lean Amount, Hard Effort or their combination) would be associated with lower adult dominance rank and lower perch position. If this prediction was supported, we aimed to further explore whether the dominance differences were mediated by differences in adult skeletal size or weight. Fourth, we predicted that greater developmental telomere attrition would be associated with lower adult dominance rank and perch position, and that developmental telomere attrition would provide greater explanatory power for adult dominance and perch position than experimental treatment alone. Fifth and finally, since in nonbreeding starlings, adult males tend to dominate females at feeding sites (Feare, 1984, Feare and Inglis, 1979), and occupy safer and more central positions in roosts (Feare, 1984), we predicted that females would have lower dominance ranks and perch positions than males in our cohort.

## Methods

### Study Subjects and Housing

Subjects were 31 hand-reared European starlings (16 male, 15 female) from eight natal families. Birds were between 11 and 13 months old at time of testing. After fledging, and prior to the current experiment, birds were housed in groups of up to 24 in an indoor ‘home’ aviary (220 × 340 cm and 220 cm high; ca. 18 °C; ca. 40% humidity; 15:9 h light:dark cycle), apart from shorter periods of individual caging to take part in behavioural experiments not described here. Each home aviary always contained complete families; hence, the quartets of birds in which dominance was measured were familiar to one another. The home aviary was provided with environmental enrichment (foraging substrate, water baths, multilevel rope perches, suspended cardboard boxes as cover), clean drinking water, and an ad libitum diet of domestic chick crumbs (Special Diets Services ‘Poultry Starter (HPS)’), cat biscuits (Royal Canin Ltd. ‘Fit32’), dried insect food (Orlux insect patée), live mealworms and fruit. Owing to the unchanging light:dark cycle of long days, the birds remained in nonbreeding condition. Birds were individually identified by unique colour ring combinations.

For the present experiments, the four birds from each family were moved for testing to one of two large experimental cages (90 × 183 cm and 183 cm high) fitted with four rope perches (horizontal perches at heights 66 cm, 121 cm, 132 cm and a sloping perch at 132–178 cm), a bath and drinker on the floor and a small table (55 × 55 cm and 45 cm high) at one end on which food bowls were placed. The cage room was maintained under the same environmental conditions as the home aviary throughout the experiment. We chose to test birds in their family groups, as this is a powerful test for the influence of early life conditions given the design of our cohort. It means that every bird's dominance was evaluated against one genetically similar bird from each of the other developmental history groups.

One bird from the original 32-bird design had died prior to the present experiment. To keep the number of competitors equal for all birds, the family from which the bird had died was supplemented with a treatment-matched bird from another family for the purposes of the experiment. The added bird had previously been run in the experiment in its own family. Excluding interactions involving this bird from the measurement of dominance and average perch position for that family made negligible difference to the dominance scores or relative perch positions of the remaining three birds. Hence, these interactions have been included for the purposes of assessing the dominance and perch position of the other birds from the three-bird family, but not for the purposes of assessing the dominance and perch position of the added bird itself.

### Developmental Manipulation

The developmental histories of the birds are described in detail elsewhere (Nettle et al., 2017). Briefly, the four siblings from each family were removed from wild nests on day 5 posthatching (where hatching is day 1). In the laboratory, they were formed into four experimental treatment groups, with one member of each natal family in each group. The first treatment manipulated the amount of food provided. In the Plenty groups, nine feeds to satiation were delivered per day via a repeating pipette. In the Lean groups, nine feeds were also delivered, but the amount of food delivered per feed was proportionately reduced; this was initially 70% of the corresponding Plenty group, as in previous studies of developmental stress (Nowicki et al., 2002), then dynamically adjusted so the growth curves of the Lean groups tracked those of the lightest nestlings in previous studies of wild starling nests. The total amount fed to the Lean groups over the course of the manipulation was 73% of that fed to the Plenty groups. The second treatment manipulated the amount of begging effort the nestlings had to exert to obtain their food: either Hard, where nestlings were stimulated to beg unrewarded for an additional amount of time approximately equivalent to the length of their actual feeds (2 min), or Easy, where no such additional begging was stimulated. The four experimental groups thus experienced the factorial combinations of the two treatments: Plenty-Easy, Plenty-Hard, Lean-Easy and Lean-Hard. The treatments continued until day 15, after which all groups were fed ad libitum until fledging, when they were moved to their home aviaries.

Both the Amount and Effort experimental treatments affected weight gain during the period they were in force. However, only Amount permanently affected adult size (Nettle et al., 2017). The Lean birds had a shorter tarsus length than the Plenty birds (Lean: mean 28.64 mm, SD 0.59; Plenty: mean 29.57 mm; SD 0.77), while the Hard and Easy birds were no different from one another (Hard: mean 29.08 mm, SD 1.01; Easy: mean 29.16 mm, SD 0.64).

To measure change in telomere length over development we compared erythrocyte telomere length (T/S ratio) measurements obtained by quantitative PCR from blood sampled at day 5 on arrival and at day 56 when the birds were independent juveniles (Nettle et al., 2017). The resulting measure, ΔTL, was standardized using the method recommended by Verhulst, Aviv, Benetos, Berenson, and Kark (2013), so that zero represents the average amount of attrition in the sample. Note that ΔTL is an inverse measure of developmental telomere attrition: a more negative ΔTL indicates greater attrition. As four assays failed, ΔTL was available for 27 of the 31 birds.

### Sexing and Biometrics

Starling nestlings are phenotypically indistinguishable by sex, and thus we were unable to counterbalance sexes within families or experimental groups. Sex was subsequently established by molecular analysis (Nettle et al., 2017). Skeletal size was established by measurement of the tarsi using digital callipers at the end of the developmental period (day 56). Measurements (mm) represent the mean of two replicate measurements of each leg. Birds were weighed using a digital balance (precision of 0.1 g) at multiple points during the experiment. For body weight, we used weight at the end of week 1 of the experiment. Results are unaffected by using weight from any other time point, or their mean.

### Experimental Procedures

In the present experiment, each family group spent 4 weeks in the experimental cage. Diet during the experiment consisted of a low-quality food (Turkey crumb), which was always available (four bowls) during weeks 0, 1 and 3, and was unpredictably removed during week 2 (see below). The diet quality was kept low during the experiment to motivate competition for a high-quality monopolizable resource (live mealworms) that was introduced for periods on alternate days to assess dominance (see Measuring dominance, below).

Week 0 consisted of cage habituation and habituation to eating mealworms from the competition bowl (see Measuring dominance, below). In week 1, Turkey crumb was available throughout; dominance and perch position were measured on alternate days as described below. In week 2, the Turkey crumb bowls were removed for periods of 6 h at unpredictable times of day, on alternate days. The purpose of this treatment was to intensify food competition, to examine whether this affected the dominance hierarchy. The protocol for week 3 was as for week 1.

### Measuring Dominance

Dominance was assessed on alternate days during weeks 1, 2 and 3, 30 min after first light. A competition bowl containing 80 g of live mealworms was placed on the table in the cage, and the subsequent 60 min of interactions were videoed using small static cameras (Vivitar DVR 785HD). The competition bowl was 9 cm tall, had a diameter of 8.5 cm, and was covered with a plastic disk with a 2.5 cm diameter hole. Thus, to access the mealworms, a bird would have to stand on the rim and probe through the hole. Only one bird could do this at a time.

**Figure 1:**
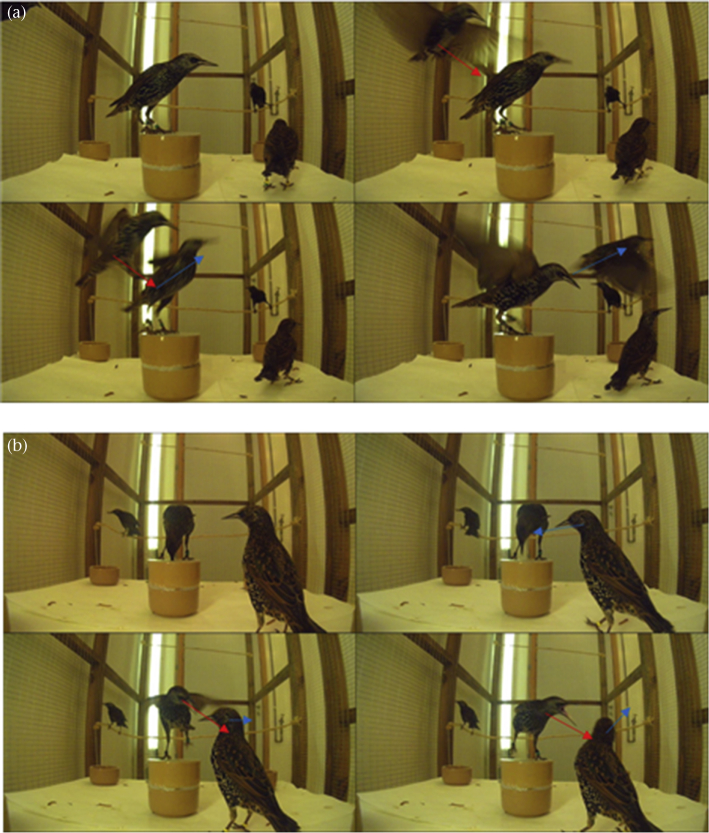
Two examples of displacement sequences from dominance videos. (a) An incoming bird (the displacer) causes an incumbent (the displaced) to leave the competition bowl. (b) The incumbent is the displacer, in that it causes a challenger approaching the bowl (the displaced) to move away.

Videos were subsequently scored by one or other of the first two authors (for inter-rater reliability, see below). Scorers were blind to the developmental treatments and sexes of the birds involved. Each incident in which one bird displaced another, or was displaced by another, at the competition bowl, was recorded. Displacements were defined as any time one bird caused another bird to move away from the bowl by moving towards the displaced bird (see Fig. 1 for examples). Thus, on this definition, a displacement occurred when the bird currently on the competition bowl prevented another bird from accessing the bowl, as well as when a bird not currently on the competition bowl ousted the incumbent. Feeding on the competition bowl was frequent, and displacements occurred at an average rate of 18.6 per hour of video. The total number of displacements was 1675, and every bird was observed as either displacer or displaced multiple times (median 81, range 19–358).

Several methods are available for quantifying dominance within groups using data from contest outcomes (Briffa et al., 2013). Our main analysis employed David's score (David, 1987, Gammell and De Vries, 2003). This measure is based on the matrix of frequencies of each individual in the quartet displacing, and being displaced by, each other. It has the advantage of controlling for the fact that different dyads may be observed different numbers of times. We computed a separate David's score for each bird in each week, and an overall David's score using all the data pooled. The separate weekly scores allowed us to assess the extent to which dominance was stable across weeks.

In addition to David's scores, we also conducted an Elo-rating procedure (as described by Neumann et al. (2011), using a starting value of 1000 and *k* = 100). Elo-rating is not based on the matrix of all interactions in a specified period. Instead, the raw sequence of interactions is used. All individuals begin with the same rating, and ratings are updated dynamically every time the individual interacts. The direction and magnitude of the change to the rating depend on the outcome and the difference in current rating between the two contestants. Elo-rating has the disadvantage of not providing a single summary number (since each individual's Elo-rating is a time series), but has the advantage of not requiring the data to be divided up into discrete time bins. There is a stability statistic associated with sets of Elo-ratings (Neumann et al., 2011) which can range from 0 (total stability) to 0.5 (no stability). This statistic was calculated separately for each family. Note that the David's scores and Elo-ratings represent the relative dominance of a bird within its natal family; its dominance vis-a-vis birds from other families was not directly evaluated.

### Perch Position

On the days between the dominance measurements, the sloping perch was filmed between 1300 and 1400 hours. The sloping perch was the highest in the cage and thus generally favoured by the birds when not feeding. The two observers scan-sampled the videos at 30 s intervals and recorded the total number of birds on the sloping perch, and the position of each bird relative to the others, i.e. highest, second, third, fourth. Scans where only a single bird or no bird was on the sloping perch were discarded. A perch position score was then calculated as 1 − (position/number of birds present). Thus, the top bird always received the highest score, and lower birds lower scores. These scores were averaged across all scans for each video, and across all videos in each week, giving an average perch position for each bird in each week. We also calculated an overall average perch position for each bird using all 3 weeks of data pooled.

In addition to this measure of average perch position, we continuously sampled the number of seconds of video each bird spent in each week as the highest-placed bird of several. The resulting times were highly correlated with the average perch positions (week 1: *r* = 0.847; week 2: *r* = 0.789; week 3: *r* = 0.856; overall: *r* = 0.879), and are not considered further.

### Inter-rater Reliability

To assess interscorer reliability for dominance, the two observers both scored three videos from each of two families, producing overall David's scores for the eight birds. The repeatability as measured by the intraclass correlation coefficient (Wolak, Fairbairn, & Paulsen, 2012) was 0.93. For perch position, the two observers both scored eight videos from across four families; the resulting intraclass correlation coefficient was 0.84. Thus, inter-rater reliability was sufficiently high.

### Statistical Analysis

Data were analysed in R (R Core Development Team, 2015; the R script and data file are provided as Supplementary material). Note that our models did not include random effects of natal family. Since David's score and average perch position were evaluated relative to other members of the same family, the means for all families were the same (exactly for David's score, and approximately for average perch position). Thus, adding a random intercept for each natal family to the model adds no information and accounts for no variation.

As we had a number of possible predictors of dominance and perch position, we used a model selection approach (Symonds & Moussalli, 2010), implemented using the R package ‘AICmodavg’ (Mazerolle, 2015). This approach is based on comparing the predictive power of alternative candidate models of the data using the Akaike information-theoretic criterion AICc. Full tables of all models compared are included in the Appendix (Tables A1–A6). For each model selection, we retained as the final set of plausible models the best-fitting (lowest-AICc) model, plus any other models whose AICc scores fell within 2 units of the best, as recommended by Symonds and Moussalli (2010). Where there was more than one model in this final set, we report the AICc weight for each one. The AICc weight can be interpreted as the relative strength of support for each of the plausible models.

In our main analyses, we considered as predictors of dominance and of average perch position the two factors we had experimentally manipulated, namely Amount and Effort, plus an important exogenous factor of interest that we had been unable to manipulate or counterbalance, namely Sex. We considered as our candidate set of models all possible models including (any of) these three factors and their possible two- and three-way interactions, plus the intercept-only model. The inclusion of the intercept-only model functions as a null hypothesis; if none of our predictors had any influence on the outcome, then the simpler intercept-only model would have the lowest AICc. Thus, there were 15 models in the main candidate set.

To explore whether any effects of developmental history or sex were mediated by skeletal size or body weight, we compared the best-fitting models identified in the main analysis described above to models including, both additionally and instead, either and both of tarsus length and weight. In the event of skeletal size and weight being important mediators or independent predictors, one or more models including these variables would have a lower AICc than the previously identified best models.

Finally, we considered whether ΔTL provided a better predictor of dominance and perch position than the experimental treatments themselves. We reran the best-fitting model identified in the main analysis using the 27 birds for whom ΔTL was available, and compared this to models including ΔTL, both additionally and instead of the other predictors of the model.

### Ethical Note

Our study adhered to the ASAB/ABS Guidelines for the Use of Animals in Research, and was approved by Newcastle University local ethical review committee. Work was conducted under U.K. Home Office project licence (number PPL 70/8089), and the removal of starlings from the wild was authorized by Natural England (licence number 20121066). Fieldwork was carried out with the permission of landowners, with number and duration of nest disturbances minimized. All nestlings began to gain weight quickly after arrival in the laboratory, suggesting rapid recovery from transport and acceptance of hand feeding (Nettle et al., 2017). There was no mortality among our hand-reared nestlings, and all but one gained weight between removal and the following evening. Nestlings were marked using coloured tape around their tarsi until large enough to be given leg rings, with no adverse effects of either method of marking. The manipulation was intended to increase developmental stress in the Lean and Hard groups. We dynamically adjusted the severity of the Amount treatment to ensure that nestling body masses remained within the natural range observed in chicks that fledged successfully from wild starling nests in our previous studies (see Developmental manipulation, above). The hand-rearing manipulations were likely to have improved the developmental experience of some or all nestlings relative to their natural fate, since natural mortality rates are often high in starlings (Feare, 1984).

Blood samples for sexing and telomere length measurement were obtained using blood samples taken from the medial metatarsal or alar vein, as reported elsewhere (Nettle et al., 2017). Combined blood sample volume was well below the prescribed limits for the percentage of total blood volume. After sample collection, blood flow was stemmed under careful observation by application of firm pressure using a cotton wool swab, and antiseptic cream applied to the puncture site to minimize risk of infection.

Stress due to catching adult birds was minimized by doing so in a darkened room using torchlight and holding birds in cloth bags for the shortest possible time. Birds received environmental enrichments (as described above) and were socially housed throughout this study. In week 2 of the experiment, when birds were food deprived for 6 h on alternate days, at least 30 min of foraging time was allowed between dawn and the beginning of deprivation, and the end of the deprivation and dusk. Wild starlings in Northumberland face darkness of up to 16 h in winter, and so are routinely unable to forage for periods longer than any experienced during the experiment. On average, the birds gained a small amount of weight during week 2 (mean 0.95 g, SD 1.17). No birds showed any adverse effects after completing the present experiment. Following the study, birds were kept for further experiments at Newcastle University.

## Results

### Stability of Dominance and Perch Position

**Figure 2:**
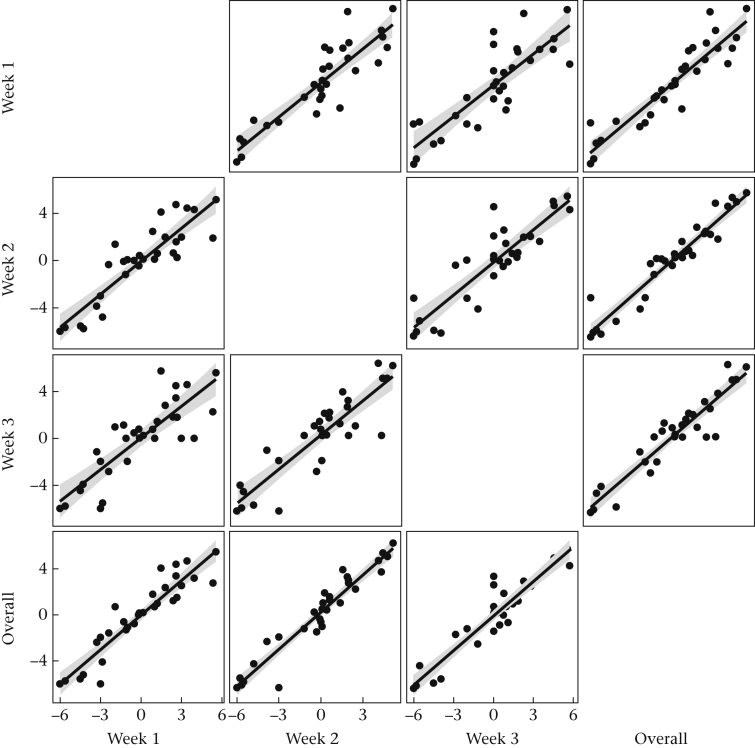
Scatterplots of dominance (David's scores) of each bird in each week of the experiment considered separately, and using the pooled data from all weeks.

The separate David's scores for dominance in each week of the experiment were strongly correlated with one another (*r* ≥ 0.839), and each was very strongly correlated with the David's score obtained by using all the data pooled together (*r* ≥ 0.928; Fig. 2). Considering all four David's scores (the three separate weekly ones, plus the overall score) as measures of the same underlying quantity, the intraclass correlation coefficient was 0.91. This suggests dominance was repeatable and stable through the experiment.

This suggestion is further confirmed by low values of the stability statistics associated with the Elo-ratings (median for the eight families = 0.088, range 0–0.147, where 0 represents perfect stability and 0.5 total instability). Birds' Elo-ratings on the final day of the experiment were highly correlated with their overall David's scores (*r* = 0.905, 95% confidence interval, CI 0.811–0.953). Given the strength of this correlation, we took the overall David's score as the measure of dominance throughout the experiment for all subsequent analyses.

**Figure 3:**
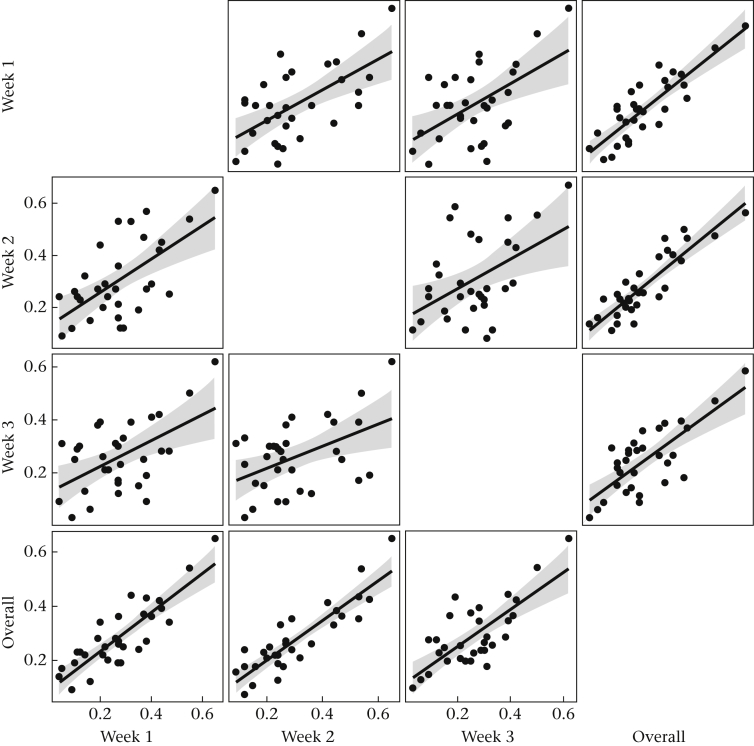
Scatterplots of average perch position of each bird in each week considered separately, and using the pooled data from all weeks.

The average perch positions also showed some stability from week to week, with interweek correlations of *r* ≥ 0.474, correlations between the overall score and each of the weekly scores of *r* ≥ 0.747 (Fig. 3) and an intraclass correlation coefficient of 0.67. Hence, we measured perch position by the overall average. Overall David's score and overall perch position were not substantially correlated with one another (*r* = 0.162, 95% CI −0.204–0.488).

### Predictors of Dominance

**Figure 4:**
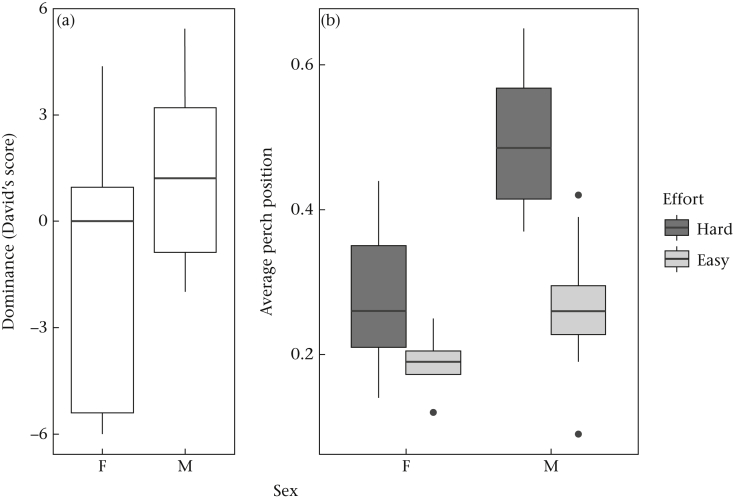
Predictors of dominance and perch position. (a) Box plots of dominance (overall David's scores) for females (F) and males (M). (b) Box plots of average perch positions for females (F) and males (M) from Easy and Hard experimental treatments. The box plots show the median and 25th and 75th percentiles; the vertical lines indicate the values within 1.5 times the interquartile range and the circles are outliers.

In the main model selection, the best model for overall David's score contained sex as the sole predictor (Table A1). This model outperformed the intercept-only model, and all those including experimental treatments, by more than 2 units of AICc. The parameter estimate for being male in this model was 2.840 (95% CI 0.595–5.085). Thus, males were generally dominant to females (Fig. 4a), and there was no support for the hypothesis that experimental treatments had any effect on dominance. We then explored the possible mediating effects of skeletal size and body weight. However, all models including tarsus length and/or body weight additionally or instead of sex performed worse than the sex-only model by at least 2 units of AICc (Table A2). Thus, neither skeletal size nor body weight was an important predictor of dominance, and the effect of sex was not explained by larger male skeletal size or higher male weight. Finally, for the 27 birds for whom ΔTL was available, we compared models including ΔTL additionally or alternatively to sex (Table A3). All models including ΔTL performed worse than the sex-only model by at least 2 units of AICc. Thus, there was no evidence to support ΔTL being an important predictor of dominance.

### Predictors of Perch Position

The main model selection for average perch position retained two models in the final set (Table A4). The first contained Sex (parameter estimate for being male 0.079, 95% CI −0.03–0.188), Effort (parameter estimate for Hard 0.084, 95% CI −0.025–0.194) and the Sex*Effort interaction (parameter estimate 0.147, 95% CI −0.008–0.301). The second model contained main effects of Sex (parameter estimate for being male 0.152, 95% CI 0.071–0.232) and Effort (parameter estimate for Hard 0.158, 95% CI 0.078–0.239) and no interaction. Thus, both models agreed that Sex and Effort were important predictors of average perch position, with males and Hard birds tending to perch higher than females and Easy birds (Fig. 4b). The two models differed only in whether these effects were additive, or whether the effect of Effort was substantially stronger for males than females. The interactive model obtained slightly higher weighting (AICc weight 0.65) than the additive model (AICc weight 0.35).

We explored whether skeletal size or body weight improved the predictive power of the best-fitting models, but all models including tarsus length or body weight in addition or instead of the previous terms were worse than the two models described above by more than 2 AICc units (Table A5).

Finally, for the 27 birds with ΔTL available, we examined the effects of entering ΔTL additionally and instead of Sex and Effort (Table A6). The best model remained that with Sex, Effort and the Sex*Effort interaction (AICc weight 0.41), and the additive model of Sex plus Effort was also retained in the final set (AICc weight 0.15). Two additional models involving ΔTL were retained in the final set: the additive model of Sex, Effort and ΔTL (AICc weight 0.23), and the same model but with the interaction of Sex and Effort (AICc weight 0.21). In these models, the average parameter estimate for ΔTL was 0.14 (95% CI −0.04–0.32). Thus, there was weak support for the possibility that ΔTL might predict perch position above and beyond the effects of Sex and Hard Effort treatment, in the direction that birds whose telomeres shortened less during development tended to sit higher than those whose telomeres shortened more, other things being equal.

## Discussion

Our repeated measurements revealed that dominance over a food resource and relative position on a sloping perch were both stable attributes of individual starlings within a given quartet over several weeks. Stabilities were equally high when we compared week 2, where we intensified competition in the group by subjecting them to periods of unpredictable food deprivation, with the other study weeks. This confirms that a single measure of dominance within a given starling group is likely to reflect an enduring ranking, as has been assumed but not demonstrated in previous studies (Feare and Inglis, 1979, Spencer et al., 2004, Swaddle and Witter, 1995, Witter and Swaddle, 1995). The Elo-ratings gave a very similar picture of dominance to the David's scores. This has also been shown to be the case for dominance data from monkey groups (Neumann et al., 2011).

Dominance was more stable over time than relative perch position. We found dominance and relative perch position to be uncorrelated with one another. This is potentially problematic for studies that amalgamate the outcomes of contests over perch position and those over food resources to obtain a single dominance measure (Swaddle & Witter, 1995).

For both dominance and relative perch position, we found clear evidence that males ranked higher than females. Previous observational studies have suggested that male starlings tend to dominate females at feeding sites (Feare, 1984, Feare and Inglis, 1979) and for position in roosts (Feare, 1984), but this is the first study to test the hypothesis quantitatively. Higher male than female dominance rank has also been observed in studies of chaffinches (Marler, 1955) and Mexican jays, *Aphelocoma wollweberi* (Barkan, Craig, Strahl, Stewart, & Brown, 1986). Interestingly, we found that the dominance of males over females was not due to their greater size or weight; in fact, neither body weight nor tarsus length was related to dominance or perch position.

Our central prediction that greater early life adversity would lead to lower adult dominance rank was not supported. There was no evidence that either of our developmental treatments, nor their interaction, had any systematic effect on adult dominance as measured in competition over a food resource. Thus, our suggestion that the behavioural phenotype we have documented as a consequence of early adversity, involving reduced dietary selectivity (Bloxham et al., 2014) and hyperphagia (Andrews et al., 2015), is due to birds that experienced greater adversity being more subordinate as adults, does not appear to be supported in this cohort of birds. This conclusion remains unaltered if we use developmental telomere attrition as the measure of early adversity, rather than experimental group: developmental telomere attrition did not predict adult dominance in competition for food.

Our findings thus differ from those of recent research in primates (Bastian et al., 2003) and rodents (Benner et al., 2015), where early insult and deprivation were shown to lead to subordinate status in adulthood. It is possible that the developmental manipulations in those studies (early maternal separation and dioxin exposure) were effectively more severe than those we used, and thus that similar effects could be found in the starling using different experimental treatments from the present ones. Nevertheless, there is a range of evidence that developmental manipulations similar to those we used are sufficiently adverse to have measurable effects on nondominance aspects of individual quality in adulthood (Boonekamp et al., 2014, De Kogel, 1997, Nowicki et al., 2002, O'Hagan et al., 2015); and indeed, one of our treatments did affect relative perch position (see below). Thus, it would be hard to argue that our treatments were simply too mild to have any impact.

Relative perch position was affected by the Effort treatment, with birds from the Hard groups perching higher on the sloping perch than those from the Easy groups. To recap, birds in the Hard treatment had to beg more each day of the developmental manipulation than birds in the Easy treatment. This was costly, in that it slowed weight gain, and was apparently stressful, as it increased developmental telomere attrition (Nettle et al., 2017). Thus, the finding that birds from the Hard treatment maintained higher rather than lower perch positions than birds from the Easy treatment is counter to our general hypothesis. There are two possible interpretations of this unpredicted finding. The first is that the high begging effort involved in the Hard treatment, as well as reducing individual quality, somehow triggered the development of greater aggression or boldness in adulthood (Drent, Carere, Groothuis, & Koolhaas, 2005), leading to defence of more desirable perch positions. However, this interpretation would be more plausible if the Hard-treatment birds were also more dominant than the Lean-treatment birds over the food resource, which they were not. Moreover, although birds frequently adjusted to each other's positions on the perch, unambiguous contests and displacements on the perch were rare.

The second possible interpretation is that the differential in desirability between the different positions on the sloping perch in our study was not sufficient to elicit strong conflict over the higher positions. Thus, what we measured with relative perch position may not have been the ability to defend a position in contests, so much as an individual preference variable. This is supported by the fact that relative perch position was uncorrelated with dominance over the food resource. Starlings generally favour the highest possible positions within a roosting tree (Feare, 1984). However, the height differences from the ground along our perch were small (46 cm of difference in height from the ground between the lower and higher end, with the lower end still 132 cm from the ground). If the higher positions had been more clearly superior in value, it is possible that the ensuing contests would have bought relative perch position into alignment with dominance rank, as was observed in Marler's (1955) study, in which contest outcomes on the perch, rather than relative perch positions, were used.

The finding that Hard birds perched higher than Easy birds could reflect behavioural compensation for their early life adversity. For example, the early adversity of the Hard birds may have led to reduced flight performance as adults. We have demonstrated such effects in a different cohort of starlings (O'Hagan et al., 2015), although we have not measured flight performance in the present cohort. Lind and Cresswell (2005) suggested that birds adjust their antipredator behaviours to their individual escape abilities, and a preference for perching higher from the ground among the Hard birds would represent an example of such as adjustment. This account might also explain why we found evidence for an interaction between Effort and Sex in predicting perch position, with Hard males favouring higher positions. Verspoor, Love, Rowland, Chin, and Williams (2007) found that the negative effect of early life adversity on flight performance was more marked in male than female starlings. We note, though, that although sex-specific effects of early life adversity have often been documented in passerine birds, which sex is more strongly affected varies across studies and outcomes (Chin et al., 2005, De Kogel, 1997, Rowland et al., 2007).

In summary, our study clearly demonstrated that the dominance ranking over food resources within starling quartets was stable over several weeks, with males ranking higher than females. It did not, however, support the hypothesis that early life adversity reduces adult dominance rank. Relative perch position in our study was also a somewhat stable attribute over time, but although males perched higher than females, perch position reflected something other than dominance rank.

## Figures and Tables

**Table A1:** Main model selection table for dominance (overall David's score)

Model	*K*	AICc	ΔAICc
Sex	3	162.01	0.00
Amount + Sex	4	164.39	2.37
Effort + Sex	4	164.46	2.44
Intercept only	2	165.99	3.98
Effort * Sex	5	166.24	4.23
Amount	3	166.29	4.28
Amount + Effort + Sex	5	167.12	5.11
Amount * Sex	5	167.22	5.21
Effort	3	167.79	5.78
Amount + Effort	4	168.32	6.30
Amount * Effort + Sex	6	168.32	6.31
Amount * Effort	5	168.81	6.80
Amount + Effort * Sex	6	169.25	7.23
Effort + Amount * Sex	6	170.18	8.17
Amount * Effort * Sex	8	174.92	12.90

*K*: number of parameters. ΔAICc: change in AICc relative to the best model.

**Table A2:** Model selection table for the effects of body weight and skeletal size on dominance (overall David’s score)

Model	*K*	AICc	ΔAICc
Sex	3	162.01	0.00
Sex + Weight	4	164.53	2.51
Sex + Tarsus	4	164.65	2.64
Weight	3	166.94	4.93
Sex + Tarsus + Weight	5	167.38	5.37
Tarsus	3	168.44	6.42

*K*: number of parameters. ΔAICc: change in AICc relative to the best model.

**Table A3:** Model selection table for the effects of developmental telomere length change (ΔTL) on dominance (overall David’s score), for the 27 birds for whom ΔTL was available

Model	*K*	AICc	ΔAICc
Sex	3	142.76	0.00
Sex + ΔTL	4	145.50	2.74
ΔTL	3	148.08	5.31

*K*: number of parameters. ΔAICc: change in AICc relative to the best model.

**Table A4:** Main model selection table for overall average perch position

Model	*K*	AICc	ΔAICc
Effort * Sex	5	−52.13	0.00
Effort + Sex	4	−50.91	1.22
Amount + Effort * Sex	6	−49.10	3.04
Amount + Effort + Sex	5	−48.31	3.82
Effort + Amount * Sex	6	−46.69	5.44
Amount * Effort + Sex	6	−45.21	6.92
Amount * Effort * Sex	8	−43.45	8.68
Amount	3	−40.42	11.71
Sex	3	−39.45	12.68
Amount + Effort	4	−39.12	13.01
Intercept only	2	−38.76	13.37
Effort	3	−37.34	14.80
Amount + Sex	4	−36.91	15.22
Amount * Effort	5	−36.46	15.67
Amount * Sex	5	−34.23	17.90

*K*: number of parameters. ΔAICc: change in AICc relative to the best model.

**Table A5:** Model selection table for the effects of body weight and skeletal size on overall average perch position

Model	*K*	AICc	ΔAICc
Sex * Effort	5	−52.13	0.00
Sex + Effort	4	−50.91	1.22
Sex * Effort + Weight	6	−49.72	2.42
Sex * Effort + Tarsus	6	−49.12	3.01
Sex * Effort + Weight + Tarsus	7	−46.35	5.78
Weight	3	−38.06	14.07
Tarsus	3	−36.30	15.83

*K*: number of parameters. ΔAICc: change in AICc relative to the best model.

**Table A6:** Model selection table for the effects of developmental telomere length change (ΔTL) on overall average perch position, for the 27 birds for whom ΔTL was available

Model	*K*	AICc	ΔAICc
Sex * Effort	5	−42.89	0.00
Sex + Effort + ΔTL	5	−41.75	1.15
Sex * Effort + ΔTL	6	−41.57	1.32
Sex + Effort	4	−40.96	1.93
Sex * ΔTL + Effort	6	−38.57	4.32
Sex + Effort * ΔTL	6	−38.56	4.33
Sex * Effort * ΔTL	9	−31.71	11.18
ΔTL	3	−27.99	14.90

*K*: number of parameters. ΔAICc: change in AICc relative to the best model.
